# Supplementary material for: The Effect of Electrical Muscle Stimulation on Muscle Mass and Balance in Older Adults with Dementia
Source: Brain Sci. 2021 Mar 7;11(3):339. doi: 10.3390/brainsci11030339 (PMC8001595; doi:10.3390/brainsci11030339)
Supplement: Supplementary file 1 [file brainsci-11-00339-s001.zip › Supplementary files/Table S1.docx]

Supplemental Table 1. Comparison between drop out and intervention complete subjects.

| Variables | Drop out  (n = 11) | Complete  (n = 21) | P value |
| --- | --- | --- | --- |
| Age, years | 89.1 ± 4.8 | 88.6 ± 5.3 | *p* = 0.7883 |
| Height, cm | 144.5 ± 4.9 | 157.1 ± 7.0 | *p* = 0.2903 |
| Body mass, kg | 46.0 ± 5.1 | 46.7 ± 5.5 | *p* = 0.7421 |
| MMSE | 12.9 ± 6.1  (6–22) | 12.9 ± 6.5  (5–25) | *p* = 0.9986 |
| FIM | 77.6 ± 28.3  (30–113) | 80.4 ± 26.7  (29–117) | *p* = 0.7885 |
| Berg Balance Score | 20.7 ± 9.6 | 22.7 ± 14.2 | *p* = 0.6874 |
| Lower limb muscle mass (kg) | 7.9 ± 1.6 | 7.8 ± 1.4 | *p* = 0.8611 |

Data are presented as the mean ± SD, (min–max).

Mini-mental state examination, MMSE; Functional independence measure, FIM
